# In Vitro Degradation of Specimens Produced from PLA/PHB by Additive Manufacturing in Simulated Conditions

**DOI:** 10.3390/polym13101542

**Published:** 2021-05-11

**Authors:** Alena Findrik Balogová, Marianna Trebuňová, Gabriela Ižaríková, Ľuboš Kaščák, Lukáš Mitrík, Jana Klímová, Jozef Feranc, Marcel Modrák, Radovan Hudák, Jozef Živčák

**Affiliations:** 1 Department of Biomedical Engineering and Measurement, Faculty of Mechanical Engineering, Technical University of Košice, 042 00 Košice, Slovakia; marianna.trebunova@tuke.sk (M.T.); lukas.mitrik@tuke.sk (L.M.); jana.klimova@tuke.sk (J.K.); marcel.modrak@student.tuke.sk (M.M.); radovan.hudak@tuke.sk (R.H.); jozef.zivcak@tuke.sk (J.Ž.); 2 Department of Applied Mathematics and Informatics, Faculty of Mechanical Engineering, Technical University of Košice, 042 00 Košice, Slovakia; gabriela.izarikova@tuke.sk; 3 Department of Computer Support of Technology, Faculty of Mechanical Engineering, Technical University of Košice, 042 00 Košice, Slovakia; lubos.kascak@tuke.sk; 4 Department of Plastics, Rubber and Fibres, Faculty of Chemical and Food Technology, Slovak University of Technology in Bratislava, 812 37 Bratislava, Slovakia; jozef.feranc@stuba.sk

**Keywords:** polylactic acid, polyhydroxybutyrate, scaffold, 3D printing, biodegradation, biomaterial

## Abstract

Biopolymers have been the most frequently studied class of materials due to their biodegradability, renewability, and sustainability. The main aim of the presented study was to evaluate degradability of the polymer material blend which was immersed in different solutions. The present study included the production of three different mixtures of polylactic acid and polyhydroxybutyrate, each with a different content of triacetin, which was used as a plasticiser. Applying 3D printing technology, two types of cylindrical specimen were produced, i.e., a solid and a porous specimen, and subjected to in vitro natural degradation. The biodegradation process ran for 195 days in three different solutions (saline, phosphate-buffered saline (PBS), and Hank’s solution) in stable conditions of 37 °C and a pH of 7.4, while the specimens were kept in an orbital motion to simulate the flow of fluids. The goal was to identify the effects of a solution type, specimen shape and material composition on the biodegradation of the materials. The monitored parameters included changes in the solution quantity absorbed by the specimens; morphological changes in the specimen structure; and mechanical properties. They were measured by compressive testing using the Inspekt5 Table Blue testing device. The experiment revealed that specimen porosity affected the absorption of the solutions. The non-triacetin materials exhibited a higher mechanical resistance to compression than the materials containing a plasticiser. The final result of the experiment indicated that the plasticiser-free specimens exhibited higher values of solution absorption, no formation of block cracks or bubbles, and the pH values of the solutions in which these materials were immersed remained neutral for the entire experiment duration; furthermore, these materials did not reduce pH values down to the alkaline range, as was the case with the solutions with the plasticiser-containing materials. Generally, in applications where high mechanical resistance, earlier degradation, and more stable conditions are required, the use of non-plasticiser materials is recommended.

## 1. Introduction

Degradation is generally defined as a loss of relevant properties of a material which develops gradually as a result of an exposure to external conditions [1]. As a matter of fact, degradation of biomaterials represents one of the biggest problems in the field of regenerative medicine. In bioengineering, the degradation may be both desired and undesired. In both cases, knowledge of the degradation kinetics is essential for the safe use of biocomponents [1]. Processes leading to the degradation of materials may generally be categorised as volumetric and surface processes. The factors affecting the volumetric degradation include an operating temperature, mechanical load and electromagnetic radiation [2].

Biomaterials, such as metals, alloys, polymers, ceramics, and composites, degrade when exposed to a strongly aggressive in vivo environment. Undesired degradation leads to the wear, corrosion, deformation, creep, fatigue, fracture and oxidation of biomaterials. Degradation may also be affected by sterilisation, to which these materials may be exposed. If a biomaterial is degraded, certain changes occur in its structure and subsequently in its properties [3]. In the regenerative medicine, there is a trend to replace natural biological materials with biomaterials where the biodegradation is desired in order to achieve a scheduled degradation of materials along with the concurrent formation of new tissues.

**Figure 1:**
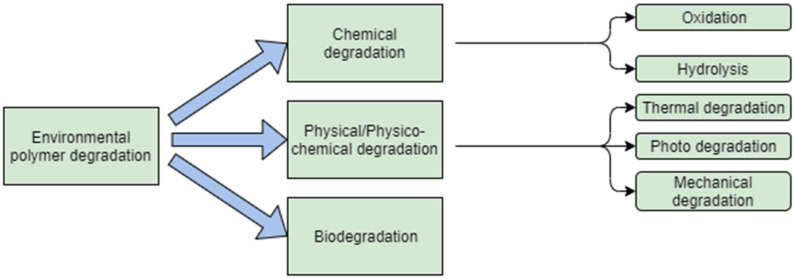
Schematic presentation of polymer degradation.

Polymers belong to the most frequently used classes of biomaterial because their properties may be modified as required by various additives, depending on a particular biological application [4]. Degradation of polymers may be induced by thermal activation, oxidation, photolysis, radiolysis, or hydrolysis (Figure 1). If the degradation is affected by the biological environment, it may also be referred to as biodegradation [5]. A plasticiser content may affect the degradation of a material. In a study performed by Pelegriny et al., they used the same type of a plasticiser as that used in the present study; it may be stated that triacetin inhibits the PLA degradation up to 45 days after the exposure, and afterwards it no longer influences the degradation process [6]. Similar results were obtained by Darabian et al.; in their study, they examined two different plasticisers. As to the absorption, the results clearly showed that a similar phenomenon has been observed in the samples containing polylactic acid (PLA) and TAC, in particular that the water absorbency increases with an increasing TAC [7].

Relevant experiments may be carried out using two types of in vitro biodegradation accelerated or natural [8]. An advantage of the accelerated degradation is that results are achieved quickly, whereas the natural degradation is often used as a control. Polymeric biomaterials may be degraded either by hydrolytic degradation in a reaction with hydrogen peroxide (H_2_O_2_), or by oxidative degradation, in which free radicals generated by a polymer directly initiate the oxidation of such a polymer [9]. This material has been subjected to the investigation because it is extensively used and due to its suitable mechanical properties and a possibility of being modified [10]. The purpose of the present study was to examine the biodegradability of polymer blends of polylactic acid and polyhydroxybutyrate (PLA/PHB), while using triacetin (TAC) as a plasticiser, in the conditions simulating a human organism. Natural biodegradation of the three types of materials with different TAC concentrations was selected for this experiment. The specimens produced from these materials by 3D printing were subjected to natural biodegradation in vitro, while the monitored parameters included the absorption, weight loss, morphological changes, and mechanical properties.

## 2. Materials and Methods

Laboratory biodegradation was facilitated using a biodegradable polymer blend of PLA/PHB, at the ratio of 85/15, containing TAC as a plasticiser in the amounts of 0%, 5% and 10% of the total volume.

### 2.1. Preparation of Materials, Specimen Designing and 3D Printing

The modelling and subsequent 3D printing were carried out using the following software products: Magics (Materialise, Gent, Belgium) for designing, modelling and structuring the specimens; Bioplotter RP (Envisiontec, Dearborn, MI, USA) and Visual Machines (Envisionec, Dearborn, MI, USA) for slicing the specimens and placing them on the platform. Prior to the 3D printing, each type of the blend material in form of a granulate with a weight of approximately 10 g was inserted into the Radwag MA50/1 moisture analyser (RADWAG, Radom, Poland) where they were dried for 60 min at a temperature of 80 °C. Drying was aimed at removing excess water molecules from the material and ensuring the durability of the material for the entire printing process. Drying was carried out due to hydrophilic properties of the PHB component [11]. Both the porous and solid specimens were designed in a cylindrical shape with a diameter of 6 mm and a height of 2 mm. The thickness of the layer represented 80% of the nozzle diameter (600 μm), i.e., 480 μm.

In both types of specimen, the individual layers were turned 90° relative to the lower layer. The distance between the centres of the individual fibres which formed the inner infill was 1.2 mm in porous specimens and 0.6 mm in solid specimens.

The 3D Bioplotter EnvisionTEC (EnvisionTEC, Dearborn, MI, USA) uses the EBB (extrusion-based bioprinting) principle, i.e., a material is pneumatically pushed out of the print head. Table 1 contains the determined parameters for 3D printing of specimens for each material type. The total number of printed specimens was 168, and they were immersed in individual solutions in groups of 7 specimens, whereas 7 porous and 7 solid specimens were used as the reference specimens.

### 2.2. Immersion of Specimens in Solutions

The experiment was carried out as natural biodegradation with three types of biodegradation media: saline solution (SS); phosphate-buffered saline (PBS); and Hank’s solution (HS). The 3D-printed specimens were immersed in these solutions in a volume of 40 mL and a pH value of 7.4, and then placed on the platform of the Orbital Shaker PSU-10i (BioSan, Rīga, Latvia), which simulated the flow of fluids during the entire experiment. The device was placed in a heated Binder incubator (Otto Bock Healthcare, Duderstadt, Germany) with a constant temperature of 37 °C.

### 2.3. Experimental Biodegradation

The materials were exposed to biodegradation for 195 days in constant conditions a temperature of 37 °C; a solution pH of 7.4, and a stirring speed of 160 rpm. The specimen weight loss and the solution pH parameters were monitored and measured at regular 15-day intervals, whereas the pH was always adjusted back to 7.4. The pH values were measured using the Mettler Toledo pH meter (Mettler-Toledo, Bratislava, Slovak Republic).

### 2.4. Weight Analysis and Calculation of Absorption

During the experiment, the specimens were regularly taken out of the solutions at 15-day intervals. The fluid on their surface was removed using the filtration paper and the specimens were weighed. The absorption percentage of the specimens was calculated using the following formula (Guo, Yang, Zhou, Chen, and Li, 2017):

(1) $$S_{w}=\frac{\left(w_{wet}-w_{dry}\right)}{w_{dry\;}}\;\times 100\;\left[\%\right]$$

The absorption of the solutions by the specimens and by the granulates is characterised as the swelling percentage S*w*, wherein W*_wet_* is the weight of a specimen on a particular day during the biodegradation; W*_dry_* is the weight of a specimen prior to biodegradation, i.e., the weight of a specimen immediately after 3D printing, before it is first immersed in a solution.

### 2.5. Monitoring of Morphological Changes

During the experiment, morphological changes were monitored using the KEYENCE VHX-5000 microscope (Keyence, Mechelen, Belgium). The specimens were examined for the fibre width and spacing, and the structure on the fibre surface. Changes in the fibre width were examined at 100× magnification on selected specimens of each material type, porous and solid, at the maximum absorption of the solution. In order to monitor all physical changes of the specimens, they were initially scanned at 30× and 50× magnifications. At 100× magnification, the fibre width and spacing were examined. The same magnification was applied when creating 3D models of the central sections of all specimens, which were then used to evaluate and graphically depict the changes in individual structures. Eventually, the surface of one fibre was scanned at 300× magnification.

### 2.6. Mechanical Testing

The mechanical compressive properties were determined using the uniaxial universal machine Inspekt5 Table Blue by Hegewald and Peschke (Nossen, Germany). The maximum load of the equipment was 5 kN. Each test specimen was fixed between the jaws and exposed to the pressure exerted by a crosshead travelling at a speed of 0.4 mm/s, while the force was applied until the specimen was completely deformed. The specimens selected for such testing were those immersed in solutions and not subjected to a microscopic analysis.

All the specimens were tested in identical testing conditions. The output graphs contain the indications of the points where a specimen exhibited the first deformation. Later on, when the compression force continued, the deformed specimens only exhibited the effects of the compression. For the purpose of calculating the strain at which the deformation of a specimen occurred, it was necessary to know not only the force magnitude, but also the specimen’s compression surface area. The surface area of a solid specimen was 2.826 × 10^−5^ m^2^. The surface area of a porous specimen was measured using the ZEISS Metrotom 1500 (Zeiss, Oberkochen, Germany). The volume of a solid specimen was 54.606 mm^3^, and the volume of a porous specimen was 42.437 mm^3^. A difference in their volumes was 12.169 mm^3^. It means that the porous specimen represented 77.71% of the solid specimen’s volume. The surface area of the porous specimen was calculated as 80% of the solid specimen’s surface area. The surface area of the porous specimen was 2.261 × 10^−5^ m^2^.

## 3. Results and Discussion

### 3.1. Evaluation of the Measured pH of Solutions

The measured pH values of the solutions indicated that the materials with different contents of a plasticiser (TAC) exhibited significant differences in pH (Table 2). Greater fluctuations of pH to the neutral or slightly alkaline ranges were observed in PLA/PHB 5TAC and PLA/PHB 10TAC immersed in SS and HS. The PLA/PHB 0TAC material immersed in HS initially induced basicity of the solution, but as the degradation advanced, the pH value stabilised. The pH value of the saline solution containing the immersed PLA/PHB 0TAC was almost always reduced (to almost the same value of approximately 6.8). The most stable pH values during the entire degradation process were observed in PBS, including the cases when the plasticiser concentrations in the basic materials were different.

### 3.2. Comparison of Solution Absorptions

**Figure 2:**
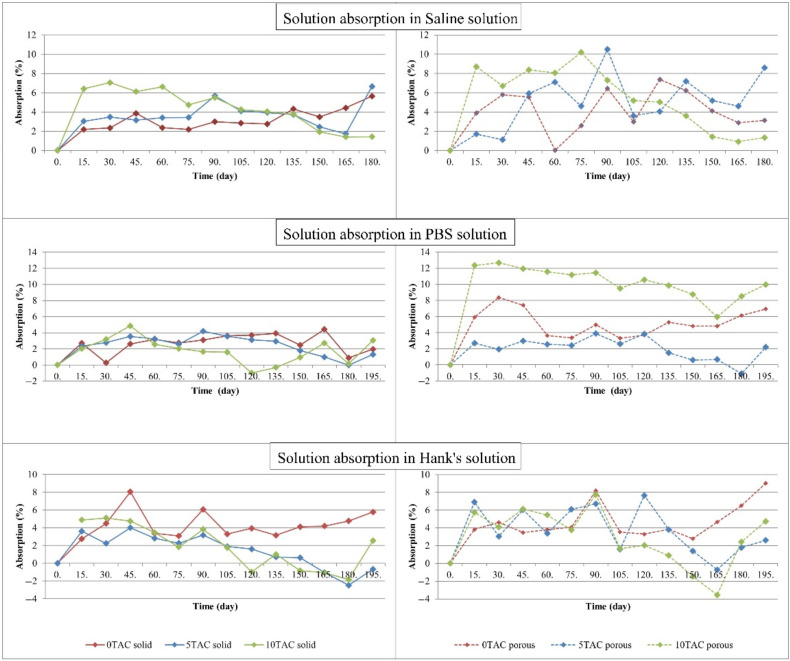
Curves of changes in the absorption of individual solutions by the specimens.

An increase in the weight of each specimen immersed in a solution was directly proportional to an increase in the absorption. The graphs in Figure 2 show a gradual increase in the absorption of the solutions. The absorption by porous specimens immersed in SS was significantly higher than the absorption by solid specimens immersed in the same solution. The average value of absorption by solid specimens was 3.8076%, while the average value of absorption by porous specimens was 5.1966%. A difference in the absorption values was also reflected in the ratio of a plasticiser content to the basic PLA/PHB material. In these specimens, the absorption of the solution increased with an increasing concentration of a plasticiser. Out of all specimens, the highest absorption value was observed in the porous specimen made of PLA/PHB 5TAC, i.e., 10.495%. The lowest absorption of SS (0.027%) was observed in the porous specimen made of PLA/PHB 0TAC.

The absorption by porous specimens immersed in PBS was significantly higher than the absorption by solid specimens immersed in the same solution. The average value of absorption of all solid specimens was 2.3423%, while the average value of absorption of all porous specimens was 5.8873%. When compared to the remaining specimens, the porous specimens with the highest contents of a plasticiser (PLA/PHB 10TAC) absorbed the highest amount of solution. The solid specimens made of the same material exhibited the lowest absorption value of all solid specimens immersed in PBS. The plasticiser-free specimens also exhibited a significant difference between the absorption values of the solid and porous specimens. The highest absorption value out of all specimens was observed in the porous specimen made of PLA/PHB 10TAC, in particular 12.676%. The lowest absorption by a specimen immersed in PBS was −1.089%, and it was observed in the porous specimen made of PLA/PHB 5TAC. This value was negative due to the fact that the weight of the specimen, as measured after it was taken out of the solution, dried and weighed on an analytical balance, was lower than its weight measured immediately after it was printed out.

When compared to the other specimens immersed in HS, the specimens without a plasticiser, both compact and porous, absorbed the highest amounts of the solution. The specimens immersed in HS exhibited the lowest average absorption value. In particular, solid specimens made of PLA/PHB 5TAC and solid specimens made of PLA/PHB 10TAC exhibited the lowest values of absorption out of all specimens. The absorption value was negative because the weight of the specimen, as measured after it was taken out of the solution, dried and weighed on an analytical balance, was lower than its weight measured immediately after it was printed out. The highest absorption out of all specimens was observed in the porous specimen made of PLA/PHB 0TAC, representing 9.02%. The lowest absorption of HS (−3.559%) was observed in the porous specimen made of PLA/PHB 10TAC.

**Figure 3:**
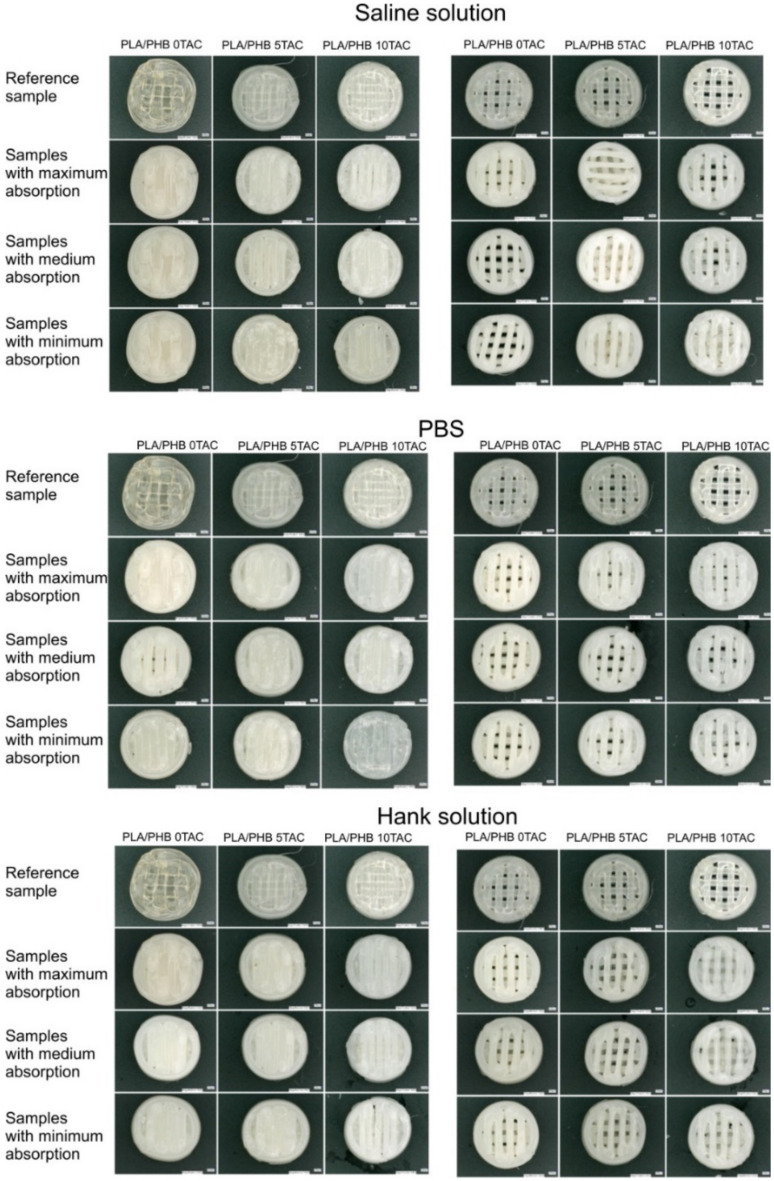
Macroscopic changes in the specimen properties after biodegradation vs. the reference specimens. The effects of absorption of solutions by the specimens on the morphological changes observed during the degradation.

The impact of the absorption on the specimens was also visible on their surface. After they were taken out of the solutions, the majority of them exhibited surface cracks. In all materials, the colour of the specimens was changed. Also, during the degradation, an additional layer of the material formed in the lower part of the specimens, probably due to the heat and the effects of a solution and a plasticiser. In some of the specimens, this resulted in a loss of porosity (Figure 3). Generally, the specimens exhibited a higher brittleness at even a simple manipulation operation.

### 3.3. Evaluation of the Measured Parameters by a Statistical Analysis

For the purpose of evaluating the types of material and solution which were used in the experiment, three specific evaluations were carried out: the effects of a specimen shape; the effects of a solution; and the effects of a plasticiser. The first step of the evaluation was testing the conditions necessary for the use of parametric or non-parametric tests.

**Figure 4:**
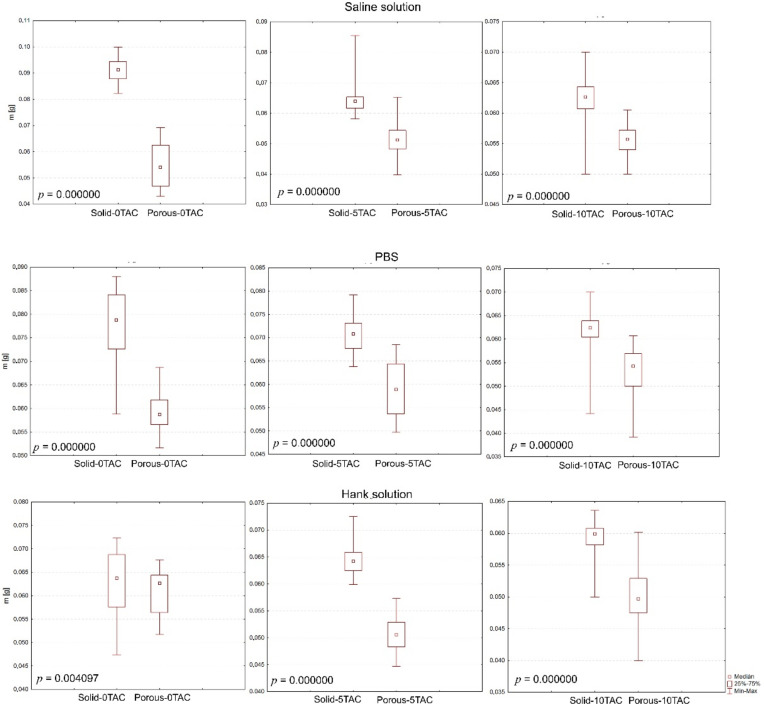
Graphical representation of the specimen shapes in terms of their statistical significance.

The evaluation of specimen shapes was carried out using the non-parametric Mann–Whitney test, which confirmed that the changes in the weights of the specimens were caused by their shapes, and that this also affected the absorption as such (Figure 4).

The evaluation of the effects of a solution type was carried out using the Kruskal–Wallis test and a post-hoc analysis as an alternative to a one-factor analysis of variance. If a *p*-value is lower than a selected significance level, the null hypothesis is rejected; this means that a difference between two medians in at least one pair, as calculated for a particular specimen, is too big to be attributable merely to a random selection. The testing revealed that the solutions affected the changes in the weights of the specimens. No statistically significant differences were observed between the solid specimens made of PLA/PHB 5TAC which were immersed in SS and those immersed in HS; or between the solid specimens made of PLA/PHB 10TAC which were immersed in SS and those immersed in PBS; or between the porous specimens made of PLA/PHB 0TAC which were immersed in PBS and those immersed in HS; or between the porous specimens made of PLA/PHB 5TAC which were immersed in SS and those immersed in HS. Statistically important differences were observed among all solid specimens made of PLA/PHB 0TAC, and among all porous specimens made of PLA/PHB 10TAC.

As for the evaluation of the effects of a plasticiser, it focused on the effects of the plasticiser contained in a material on identical solutions and identical specimen shapes; the total number of the performed tests was 6. The evaluation of the effects of a plasticiser was carried out using the Kruskal–Wallis test, which confirmed again that the plasticiser (0%, 5%, and 10%) affected the changes in individual weights. No statistically significant differences were observed for the porous specimens made of PLA/PHB 0TAC or PLA/PHB 10TAC which were immersed in SS; or for the porous specimens made of PLA/PHB 0TAC or PLA/PHB 5TAC which were immersed in PBS. However, statistically significant differences were observed for this particular solution in all solid specimens immersed in PBS. As for the solid specimens made of PLA/PHB 0TAC or PLA/PHB 5TAC which were immersed in HS and the porous specimens made of PLA/PHB 5TAC or PLA/PHB 10TAC which were immersed in HS, there were no statistically significant differences.

### 3.4. Morphological Changes in the Specimens

**Figure 5:**
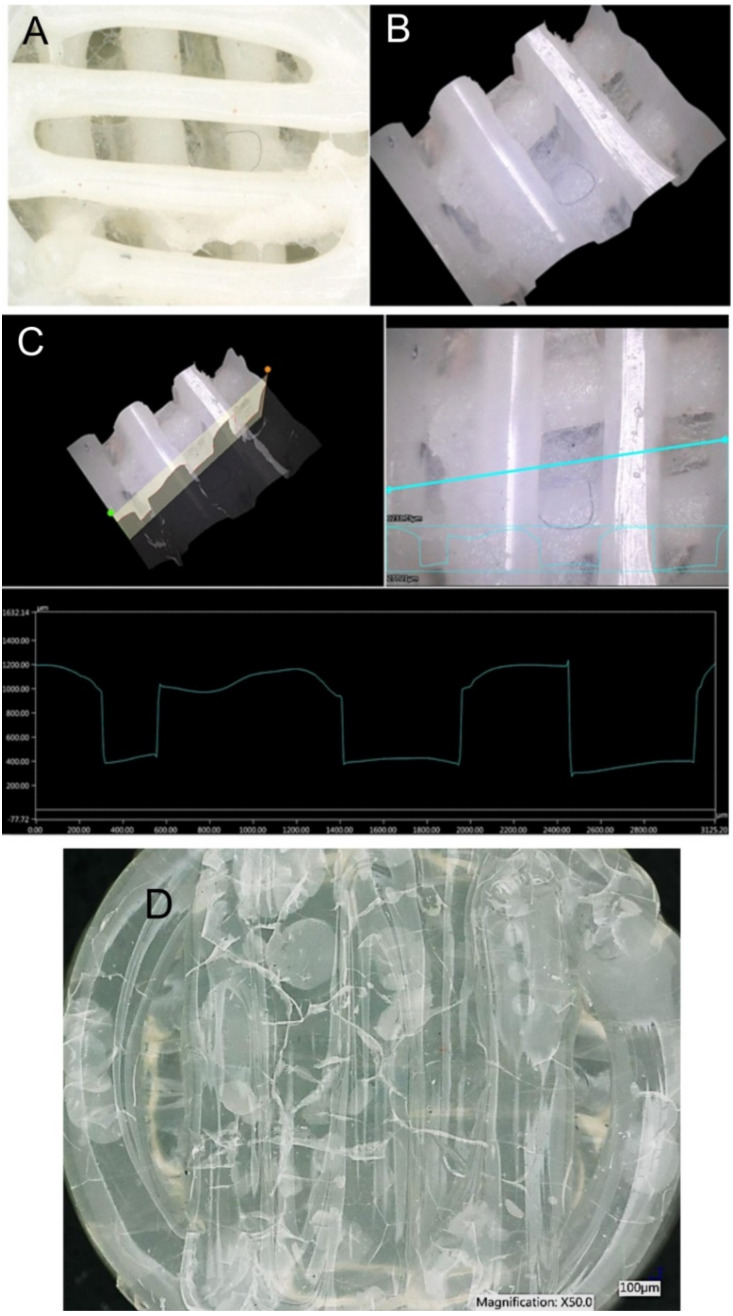
**A**–A macroscopic image of a specimen with a visible film formed in its lower part. **B**–The created 3D model of the central part of the specimen; it is visible that the specimen is no longer porous. **C**–A 3D analysis of the changes in the specimen surface. **D**–A polylactic acid and polyhydroxybutyrate (PLA/PHB) 10TAC specimen after degradation in PBS. Visible presence of bubbles and block cracks on the surface of the specimen.

In terms of morphological changes, the focus of the monitoring was on the changes in the surface of the individual specimens and on the changes in the width of the extruded fibres. The most significant change in the fibre width was observed in the solid specimens made of PLA/PHB 0TAC, which exhibited a fibre width after degradation as low as approximately 1 mm. An increased fibre width was observed in the porous specimens made of PLA/PHB 0TAC; this material did not contain a plasticiser and was immersed in all three solutions. The only negative value, i.e., a decrease in the fibre width, was observed in the porous specimen made of PLA/PHB 5TAC, which degraded in SS (Table 3). This decrease in the fibre width might have been caused by the fact that the specimen lost its porosity after degradation because a film of a material was formed in its lower layer. This film was probably formed from a certain portion of the material released from the individual fibres of the specimen, and that is why the width of the fibres decreased. A macroscopic image of the specimen shows a visible film formed in the lower part of the specimen (Figure 5A). A 3D image of the central part was subsequently used to create the cross-sections of individual parts (Figure 5B). The graph represents the development of the surface. If the specimen was porous and with no film, the lower value in the graph would be 0 μm. However, this graph of the surface development confirms the presence of the film at the height of approximately 400 µm (Figure 5C).

The distance between the individual fibres in solid specimens should be 0 µm, and in porous specimens it should be 600 µm. This means that every value that was lower than 600 μm in porous specimens indicates an increase in the fibre width and a consequent decrease in a distance between two fibres. This indicates that the solution was absorbed by the fibres (Table 3). All negative values contained in Table 3 indicate the reduced distances between the fibres. A positive value of the difference means that the distance between the fibres increased. The difference represents the value after biodegradation in the solutions relative to the specimens prior to biodegradation.

Degradation of specimens was also confirmed in one more aspect. As there were three types of material and three types of solution, it was assumed that each material may behave differently in a different solution, even after a specimen is taken out of the solution. In our case, the solid specimens made of PLA/PHB 0TAC which were immersed in the saline solution and the solid specimens made of PLA/PHB 10TAC which were immersed in PBS exhibited the formation of block cracks and bubbles (Figure 5D). These changes were not visible while the specimens were still immersed in the solutions. After they were taken out and dried, their surface exhibited some block cracks and bubble-like lighter areas visible to the naked eye.

### 3.5. Mechanical Testing

Based on the values at which the specimens exhibited deformation, a general conclusion may be made that the plasticiser-free specimens were more resistant to compression, regardless of the solution they were immersed in.

#### 3.5.1. Saline Solution

**Figure 6:**
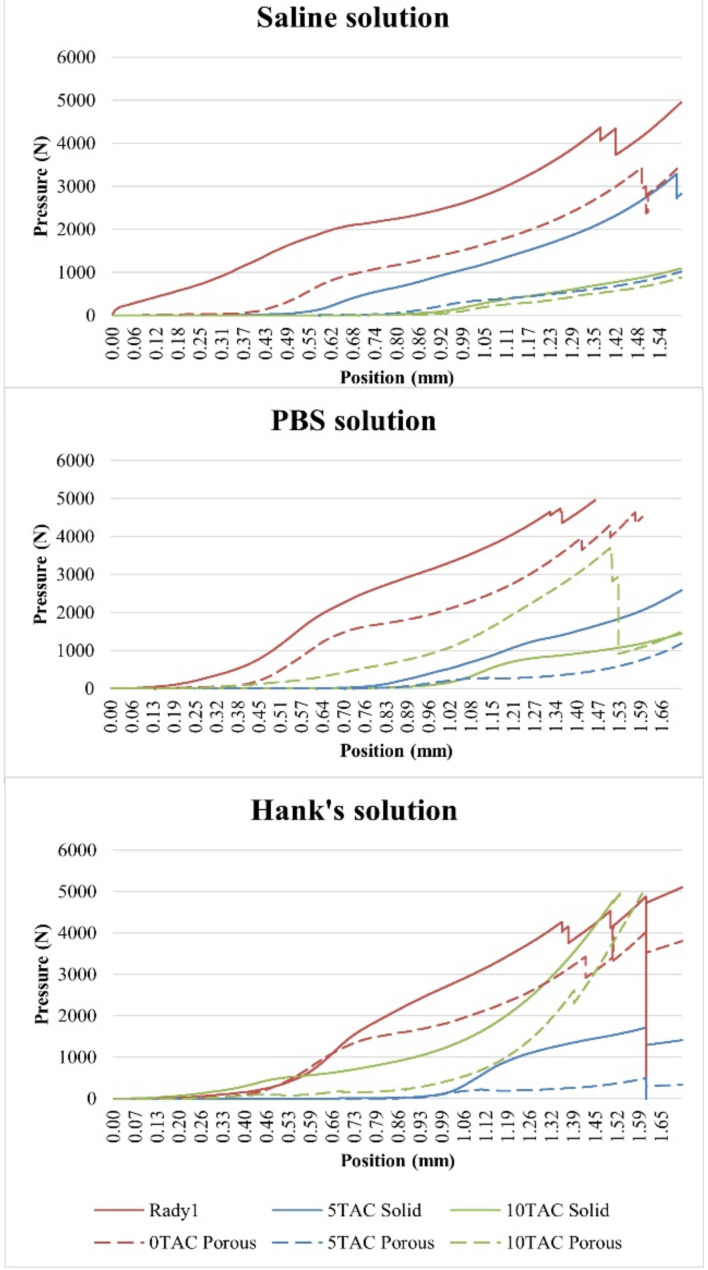
Curves of the average values measured during the mechanical compression testing of the individual materials after biodegradation.

The highest strain value was observed for the solid specimens without a plasticiser. Even though the strain value of the porous specimen made of PLA/PHB 0TAC was significantly lower than the strain of the solid specimens made of the same material, this value was always higher than that of the porous specimens containing a plasticiser. As for the specimens containing a plasticiser, the solid specimens exhibited deformation earlier than the porous ones. This might have been caused by the presence of block cracks in the specimens after drying. With regard to the specimens immersed in the saline solution, it is possible to state that as a concentration of the plasticiser increased, the value of strain at which the deformation occurred decreased (Figure 6).

#### 3.5.2. Phosphate-Buffered Saline (PBS)

The highest value of the strain was observed for the porous specimens that did not contain a plasticiser. Although the strain value of the solid specimen made of PLA/PHB 0TAC was lower than the strain of the porous specimens made of the same material, this value was always higher than the value measured in the solid specimens containing a plasticiser. As for the specimens containing the plasticiser, the solid specimens exhibited deformation earlier than the porous ones. With the use of this type of solution, it was observed that the specimens made of PLA/PHB 5TAC exhibited deformation earlier than those made of PLA/PHB 10TAC. Contrary to the finding made with SS, it was not confirmed that with an increasing concentration of a plasticiser the value of strain at which the deformation occurs decreased, because the lowest value of strain at which the specimen cracked was observed in the specimens made of PLA/PHB 5TAC (Figure 6).

#### 3.5.3. Hank’s Solution

As for the specimens that did not contain a plasticiser, the porous specimens were more resistant. The highest value of strain at which a porous specimen broke was observed for the specimen made of PLA/PHB 0TA, i.e., without a plasticiser; all the remaining porous specimens containing a plasticiser exhibited deformation at earlier times. As for the specimens containing a plasticiser, the porous specimens were deformed earlier than the solid ones. With the use of this particular solution, the PLA/PHB 5TAC material exhibited deformation earlier than the specimens made of PLA/PHB 10TA. Therefore, unlike with SS, with HS it was not confirmed that with an increasing concentration of a plasticiser the strain at which the specimen was deformed decreased, because the lowest strain value at which the deformation occurred was observed for the specimens made of PLA/PHB 5TAC (Figure 6).

## 4. Discussion

In the present case study, it is rather problematic to discuss the results obtained with other authors, because there are only a few scientists who have examined biodegradation of the identical mixtures of materials by applying the same method. The PLA material is comprehensively studied as it is widely used as a green polymer in many areas [12]. Several studies have dealt with biodegradation of the individual components separately while concentrating mainly on the strength properties of these polymers. In 1995, relevant testing was performed by Lianlai Zhang et al., who examined the mixability of different polymer blends, including the PLA/PHB mixture, and their properties, which were impaired by hydrolytic degradation. The study confirmed that this material might be used in future medical applications thanks to its biodegradation properties [13]. This conclusion correlates with the obtained results which confirmed that this material decomposes in a water environment. Freier et al. also examined biodegradation in vitro in a buffer solution and in a solution containing pancreatin extracted from rats [14]. In standard conditions (37 °C; pH 7.4), the molecular mass of pure PHB decreased by one half, and in the PLA by one third. The authors also observed slower degradation following the addition of hydrophobic plasticisers triethyl citrate (TEC) and butyryl trihexyl citrate (BTHC). The experiment revealed that the greatest weight loss was observed particularly for the mixture with the highest TAC content; therefore, we can state that some plasticisers may significantly affect the speed of biodegradation. Also, the accelerating effect of pancreatin on PHB degradation was confirmed as the degradation speed exhibited a three-fold increase. Based on the study results, it is possible to state that despite the initial resistance of the PHB material to low pH for a sufficiently long period of time, it is eventually capable of complete decomposition in an organism.

Several studies have dealt with the preparation of scaffolds from the composite materials containing PHB and ceramics while examining their biodegradation in simulated body fluids. Wang et al. created the hydroxyapatite (HA) material, which was mixed with poly (3-hydroxybutyrate) (PHB), poly (3-hydroxybutyrate-co-3-hydroxyhexanoate) (PHBHHx) and a plasticiser, and the scaffolds made this material were monitored for 50 days while being immersed in simulated body fluid (SBF) solution. They measured the weight loss and morphological changes in the materials; after 50 days of degradation in vitro, PHB exhibited almost no changes and PHBHHx lost 7% of its weight [15]. A similar study was also performed by Ni and Wang; they monitored a PHB/HA mixture and evaluated its biodegradation by applying scanning electron microscopy (SEM) and dynamic mechanical analysis (DMA) [16]. The scaffolds produced were immersed in SBF solution and monitored at 37 °C for 2 weeks, 1 month, 2 months and 4 months. The SEM revealed that mineral crystals grew on all HA/PHB composite specimens after 1 day of being immersed in SBF; after 7 days, the mineral layer grew more intensively and the surface of the mineral layer was significantly smoother. The morphology of the mineral layer, which was formed after the immersion in SBF for 2, 4 and 8 weeks, was similar to the morphology of the mineral layer formed after the immersion in SBF for 7 days. A dynamic mechanical analysis (DMA) revealed that the storage module (E’) of the HA/PHB composite was initially increasing over time while it was immersed in SBF, as a result of the formation of apatite on the surface of the composite, but it decreased after a prolonged immersion in SBF; this indicated the degradation of the composite.

Three-dimensional printing was applied by Barbeck et al. in order to produce scaffolds made of pure PLA, PLA/G5 bioglass with a plasticiser, and polyethylene glycol (PEG). The specimens were immersed in SBF at 37 °C for 8 weeks, and their weight loss and mechanical properties were monitored [17]. A response of the connective tissues to the presence of a scaffold was also monitored. Its mechanical properties impaired over time during the degradation. The same finding was also confirmed in our study. Mechanical compressive properties were better in the scaffolds made of PLA/G5 than in those made of pure PLA.

Biodegradation of a combination of PLA and silica glass was also investigated by El-Kady et al. They monitored the biological activity of scaffolds immersed in SBF at a constant temperature and a constant pH while the specimens were incubated for 30 days [18]. They monitored the weight loss, absorption of water, and changes in pH. A HA layer formed on the surface of the scaffolds; it was scanned using the scanning electron microscope SEM and subjected to an energy-dispersive X-ray analysis.

Vergnol et al. monitored the biodegradation of a combination of poly(L, DL)lactide (P (L, DL) LA) and particles of bioglass 45S5. Similar to our study, they measured the absorption of PBS on the basis of the weight loss; the microscopic properties were measured by tomography; and mechanical properties were tested by compressive testing [19]. The authors observed that after 6 months of degradation, the specimens lost 35% of their original weight, and the tomography revealed the presence of cracks in these materials. The conclusion was that the mechanical properties of such specimens were worse than those of the reference specimens.

Scaffolds containing PLA and PHB have a promising potential for the applications with animal cells; that is why these biomaterials are currently subjected to the extensive research in their biodegradability potential. A paper by Li et al. described the testing of various PHB copolymers by applying the method of accelerated degradation in SBF at 37 °C. It was observed that poly(3-hydroxybutyrate-co-3-hydroxyhexanoate) maleate copolymer lost 21.4% of its original weight after being immersed in solution for 21 weeks [20]. In the study by Boskhomdzhiev et al., they monitored the rate of in vitro biodegradation of PHB in the presence of lipase as well as in vivo degradation after being implanted in an animal tissue. They confirmed that the biodegradation of PHB includes a polymeric hydrolysis as well as enzymatic degradation [21].

Guo et al. investigated a possibility of characterising the biologically degradable porous scaffolds made of PLA which were intended to be used in tissue bioengineering; the investigation was carried out by applying the selective enzymatic degradation. The porous morphology and the properties of such scaffolds were examined in order to identify whether the microstructure of the biomimetic extracellular matrix is suitable for the cell proliferation and differentiation. PLA scaffolds degraded into harmless products in the SBF solution at a low rate of degradation. The weight loss after 8 months of the in vitro degradation represented 80%. It was also observed that PLA scaffolds supported the cellular interconnection and growth. These findings confirmed that the selective enzymatic degradation may be applied in the production of scaffolds [22].

Biomaterials made of PLA and PHB are often subjected to experiments aimed at discovering potential applications in food packaging and their subsequent biodegradation in the composting conditions. Biodegradation and physical evaluation of the packaging materials made of PHB were also discussed in the paper by Bucci et al. The properties of such packaging materials were assessed by physical tests and compared to polypropylene (PP). The results showed that these packaging materials acted as an effective barrier against light and humidity. Biological degradation began after 60 days in compost, where the packaging materials almost completely decomposed [23].

Savenkova et al. investigated the effects of plasticisers on the physical and biodegradation properties of PHB-based films. They applied accelerated degradation testing (ADT) at a temperature of 25 °C in the microbially active soil layers while the specimens were left in the soil for 30 days. Several bacteria species were able to degrade this material, and the resultant finding was that as the concentration of plasticisers increased, the rate of biodegradation proportionally decreased. Even though the biodegradation which was performed in the study by Savenkova et al. was carried out in compost, it is possible that the presence of plasticisers had a negative effect on the bacteria contained therein [24].

A similar idea was experimentally investigated by Wisuda Pattanasuttichonlakul et al. In their paper, they described the examination of biodegradation using ultraviolet C (UV-C) light and the subsequent placement of specimens in the soil containing microbiological organisms collected from the waste water sledge. They observed that PLA exhibited good degradation properties as it completely decomposed in the soil [25].

A PLA/PHB mixture was investigated by Dvořáčková et al. for biodegradability in a water environment using the sledge from a waste water treatment plant, in particular the mesophilic and thermophilic anaerobic sledge. The study confirmed that PLA did not decompose in the mesophilic conditions, whereas in a thermophilic environment it degraded in more than 80%. After 60 days of abiotic hydrolysis of the PLA/PHB mixture, degradation represented 23.8%. This has proved that hydrolytic enzymes affect a biodegradation degree [26]. All the aforementioned authors achieved similar results, which indicate that PLA/PHB-based materials may be used in the development of various medical devices or food packaging materials due to their biodegradation properties. In further experimental research, the authors of this paper will perform chemical analyses of this material after degradation and degradation products in solution.

## 5. Conclusions

In our experiment, we found out that the examined material gradually degraded in the in vitro conditions; this biomaterial is, therefore, suitable for further investigation. The most suitable specimen was a solid specimen with a 5% content of a plasticiser, and it exhibited the average absorption value of 1.443 ± 0.001% after 195 days of being immersed in HS. As for the porous specimens, the lowest value of absorption (2.059 ± 0.001%) was observed in the experiment with the PLA/PHB 5TAC material immersed in PBS solution. On the basis of the performed tests it is possible to conclude that the difference between the absorption values was affected by the differences in the compositions of the biodegradation media. A statistical analysis revealed that a specimen shape, type of a degradation medium, and a plasticiser content affected the changes in the specimen weight and consequently also the changes in absorption. The evaluation of morphological changes in the specimens revealed the formation of block cracks in the specimens containing a TAC plasticiser. Mechanical compression testing of the specimens resulted in the finding that deformation of the plasticiser-free specimens began at the latest time. This means that they are mechanically more resistant to compression than the specimens containing a plasticiser.

The final result of the experiment was the finding that even though the specimens without a plasticiser exhibited higher values of absorption of a respective solution, they were not present with any block cracks or bubbles. Also, the pH values of the solutions in which they were immersed were neutral for the entire duration of the experiment.

## Figures and Tables

**Table 1:** Print parameters of individual materials used for the production of specimens (temperature curve, pressure, and speed).

TAC Content	0TAC	5TAC	10TAC
Heating temperature and time	230 °C/2 min 200 °C/3 min 190 °C/2 min 185 °C/5 min	230 °C/2 min 200 °C/3 min 190 °C/2 min	230 °C/5 min 200 °C/2 min
Pressure	Porous: 7.7 bar Solid: 7.1 bar	Porous: 6.5 bar Solid: 7.5 bar	Porous: 6.7 bar Solid: 7.2 bar
Print speed	Porous: 1.3–1.6 mm/s Solid: 1.9–2.2 mm/s	Porous: 2.5–2.7 mm/s Solid: 2.1–2.3 mm/s	Porous: 3.5–3.8 mm/s Solid: 3.2–3.5 mm/s

**Table 2:** Changes in pH values during the experiment (saline solution—SS, Hank’s solution—HS, PBS—phosphate-buffered saline).

	Day:	15	30	45	60	75	90	105	120	135	150	165	180	195
	TAC													
SS	0	6.85	6.95	6.88	6.95	6.86	6.86	6.91	6.83	6.85	6.95	6.41	6.47	6.51
	5	7.22	7.26	7.16	7.08	7.21	7.12	4.45	5.14	4.11	4.38	4.67	4.01	4.06
	10	7.23	7.13	6.50	7.00	6.55	5.41	4.63	5.08	4.51	4.63	4.77	4.87	4.99
PBS	0	7.18	7.30	7.28	7.35	7.34	7.35	7.19	7.31	7.30	7.43	7.42	7.41	7.38
	5	7.11	7.32	7.32	7.36	7.36	7.33	7.16	7.05	6.97	7.17	7.16	7.06	7.12
	10	7.08	7.34	7.29	7.31	7.26	7.19	6.89	7.10	6.85	7.07	7.14	7.20	7.22
HS	0	7.77	7.46	7.48	7.59	7.44	7.37	7.69	7.41	7.38	7.39	7.39	7.31	7.34
	5	7.62	7.34	7.48	7.37	7.43	7.26	5.03	5.31	4.5	4.62	4.62	4.47	4.51
	10	7.40	7.37	7.45	6.90	6.51	6.05	4.91	5.07	4.51	4.63	4.59	4.25	4.38

**Table 3:** Measured values of a fibre width and a distance between the two fibres for the individual materials immersed in different solutions.

Maximum Fibre Width [µm]							
		0TAC	Difference	5TAC	Difference	10TAC	Difference
Saline solution	Solid	1068	468	797	197	686	86
	Porous	840	240	551	−49	610	10
PBS	Solid	1061	461	803	203	825	225
	Porous	762	162	675	75	777	177
Hank’s solution	Solid	677	77	740	140	718	118
	Porous	810	210	623	23	716	116
**Maximum distance between 2 fibres [µm]**							
		**0TAC**	**Difference**	**5TAC**	**Difference**	**10TAC**	**Difference**
Saline solution	Solid					158	158
	Porous	464	−136	571	−29	534	−66
PBS	Solid						
	Porous	486	−116	493	−107	451	−149
Hank’s solution	Solid	122	122			61	61
	Porous	429	−171	603	3	436	−164

## Data Availability

The data presented in this study are available on request from the corresponding author.
